# Phylogeographical structure and demographic expansion in the endemic alpine stream salamander (Hynobiidae: *Batrachuperus*) of the Qinling Mountains

**DOI:** 10.1038/s41598-017-01799-w

**Published:** 2017-05-12

**Authors:** Zu-Shi Huang, Feng-Lan Yu, Hui-Sheng Gong, Yan-Ling Song, Zhi-Gao Zeng, Qiong Zhang

**Affiliations:** 1 State Key Laboratory of Integrated Pest Management & Key Laboratory of Animal Ecology and Conservation Biology, Institute of Zoology, Chinese Academy of Sciences, Beijing, 100101 China; 2 Foping National Nature Reserve, Foping, 723400 Shaanxi China

## Abstract

The Qinling Mountains of China provide an excellent study area for assessing the effect of Pleistocene climatic oscillations and paleogeological events on intraspecific diversification. To assess genetic diversity of an endemic stream salamander, *Batrachuperus tibetanus*, for its conservation, a phylogeographical survey was performed based on mitochondrial DNA and morphological data. The mitochondrial data revealed three lineages of *B*. *tibetanus* in the Qinling Mountains. A lineage present in the northwestern Qinling Mountains groups with the Tibet lineage of *B*. *tibetanus*, and the remaining Qinling populations are eastern and western lineages that separated ~3–4 million years ago (Ma). The eastern and western Qinling lineage delineation is supported by three morphological variables (snout length, eye diameter and axilla-groin length). The divergence of the two major lineages was likely caused by orogenesis of the Qinling Mountains during the late Cenozoic, and the two lineages were subsequently affected at different levels by Pleistocene climatic oscillations showing different signals of demographic expansion. A large suitable area of *B*. *tibetanus* through the Qinling Mountains since the last glacial maximum (LGM) indicated the adaptation of this species to the climatic changes. However, low genetic diversity within populations indicate the urgency of preserving the vulnerable populations and endemic lineages.

## Introduction

Modern patterns of intraspecific genetic diversity presumably reflect cyclical Pleistocene climatic oscillations and Meso-Cenozoic intracontinental orogenesis. Most notably, cyclical Pleistocene climatic oscillations have caused extinction and repeated changes in the distributions of those species that survived in Europe and North America^1–3^. In China, the continuing uplift of the Tibet Plateau, the highest plateau on earth, and associated climatic fluctuations appear to be the most important factors driving the geographic genetic diversification of populations in southwest China^4–6^, which is one of the most important biodiversity hot-spots in the world. Similar to the Tibet Plateau, the geographically adjacent Qinling Mountains provide another excellent area of China for evolutionary study of species. However, the Qinling Mountains have been less studied than the Tibet Plateau.

The Qinling Mountains, formed during the Mesozoic and Cenozoic periods^7, 8^, are a major mountain range extending over 1600 km from the east to the west in southern Shaanxi province of China. To the west is the northern edge of the Tibetan Plateau, while to the east are the plain and hilly areas of eastern China. The Qinling Mountains are an important natural barrier for the northern temperate regions and the semi-tropical southern ones, and form the watershed for the Yangtze and Yellow Rivers. Taibai Mountain is the highest peak of the Qinling Mountains (3767 m) and considered to be the center of glaciation in the Qinling Mountains with limited Late Pleistocene ice-cover^9–13^. The Qinling Mountains are located in an important geographical position with complex topography and variable climates and habitats, contributing to the biodiversity of the Eastern Asian flora and fauna by harboring many endemic species^14^, such as the giant panda (*Ailuropoda melanoleuca*) and the golden takin (*Budorcas taxicolor bedfordi*). In recent years, phylogeographical studies revealed that the Qinling Mountains probably served both as a major barrier and glacial refugium for plants and animals^15–21^.

The alpine stream salamander *Batrachuperus tibetanus* (family Hynobiidae) is endemic to China. It usually inhabits fast-flowing montane streams in the eastern edge of the Tibetan Plateau and the Qinling and Daba Mountains from 1600 to 4300 m above sea level^22^. It is threatened by habitat loss and over-hunting for traditional Chinese medicine and food, and thus listed as a vulnerable species in the IUCN (the International Union for Conservation of Nature) Red List category^23^. The stream salamanders are not only indicators of overall environmental health as are other amphibians^24^, but also are ideal species for evolutionary research on account of potentially strong phylogeographical signal and ease of sampling. Due to weak dispersal ability, stream salamanders tend to be highly structured genetically over short geographical distances^25, 26^ and retain high-resolution genetic signals of historical processes that have shaped current phylogeographical patterns^26^. The closely related species of stream salamanders occurring at different elevations in southwest China may have experienced different evolutionary histories driven by topography and responses to glaciation^26^. For example, the stream salamanders living in the western high-elevation regions seem to have experienced colonization and population declines due to the last glaciation, while those in the eastern low-elevation regions appear to have experienced fragmentation during the extensive glacial period^26^. However, it is unclear how the population-genetic diversity of *B*. *tibetanus* in the Qinling Mountains was structured by historical processes and affected by topography, glaciation and drainage.

Interestingly, there are two completely different phylogeographical patterns reported in the Qinling Mountains, i.e. north-south break^15, 16^ and west-east or west-central-east breaks^19–21, 27–29^, indicating that Qinling Mountains may have played different roles in evolutionary history of different species in this region. The current genetic patterns of stream-associated amphibians may have been shaped by drainage systems^30–34^. Thus, drainage systems ascribed to the Yangtze River and Yellow River may have influenced the distribution and population structure of *B*. *tibetanus* in the Qinling Mountains, and then generated a north-south break. This hypothesis appears to be supported by the distinct climates and habitats at both slopes of the Qinling Mountains that serve as the boundary of the Palearctic and Oriental realms. However, the Qinling Mountains could serve as a major barrier to generate the north-south break for species generally distributed in the low altitude localities of the northern and southern sides (e.g. <1200 m above sea level)^15, 16^. In contrast, for species generally distributed in the high altitude localities of the Qinling Mountains, tectonic changes, Pleistocene climatic fluctuations and population history likely have resulted in the remarkable west-east and/or west-central-east breaks^20, 21, 29^, given that these species generally have weak dispersal abilities. The stream salamanders are generally distributed in the montane streams in the Qinling Mountains (>1200 m above sea level), and thus possibly affected by orogenesis of the Qinling Mountains and Pleistocene climatic oscillations^9–13^ resulting in a west-east break.

**Figure 1:**
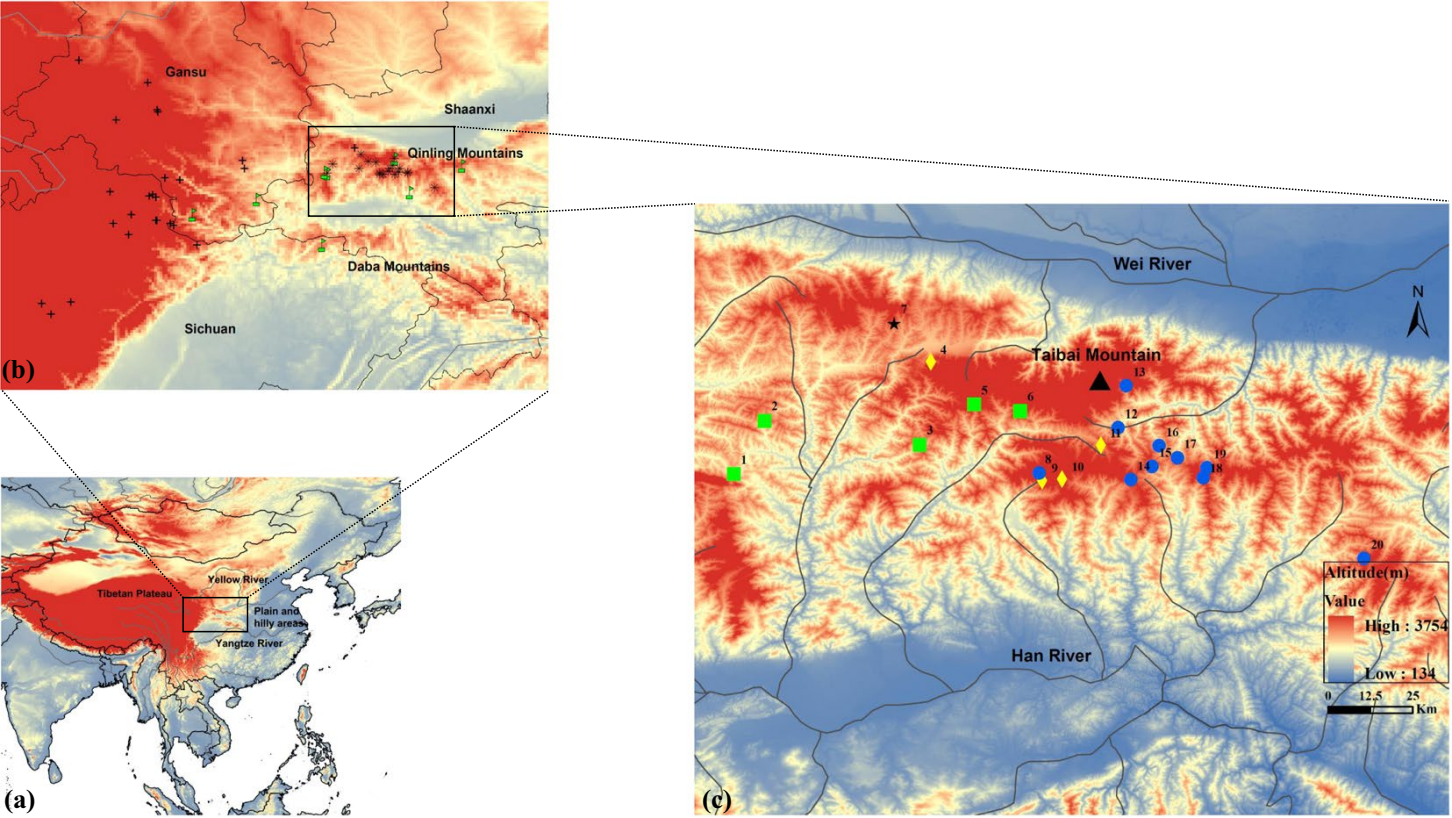
Geographical distribution of *B*. *tibetanus* in China and sampling locations in the Qinling Mountains used in this study. (**a**) Geographical distribution of *B*. *tibetanus* across its main distribution range in China. (**b**) Distribution of the Tibet and eastern lineages of *B*. *tibetanus*. The Tibet lineage of *B*. *tibetanus* is labelled by cross including 24 locations from prior study^25^ and the QLC population of this study, and the eastern lineage is labelled by school (8 locations from prior study) and asterisk (19 of 20 sampling locations of this study). The eastern lineage of *B*. *tibetanus* is previously described as *B*. *taibaiensis* ^35^. (**c**) Sampling locations in the Qinling Mountains used in this study. The two major population lineages are labelled by green squares and blue circles, respectively. The yellow diamonds indicate populations possessing haplotypes from both lineages. The QLC population is labelled by a black star. The numbers indicate different populations (see Table 1). The optimal number of population groups is three revealed by the Spatial Analysis of Molecular Variance (SAMOVA). The western group consists of the population 1–6, the eastern group contains the population 8–20, and the third group contains only the QLC population. The main rivers in the distribution area are shown by lines, and the Taibai Mountain (3767 m) is labelled by a black triangle. The map was generated using ArcGIS 9.3 (http://www.esri.com/software/arcgis) and Adobe Illustrator CS4 v14.0.0 (Adobe Systems Inc., San Francisco, CA). The Digital Elevation Model (DEM) for Fig. 1c was downloaded from the website, which was provided by Data Center for Resources and Environmental Sciences, Chinese Academy of Sciences (RESDC) (http://www.resdc.cn).

Previously, the stream salamanders distributed in the Qinling Mountains were described as a new species *B*. *taibaiensis*, which is distinguished from geographically neighboring species, *B*. *tibetanus*, by its large size (adult males over 217 mm in total length) and the inverted V-shaped vomerine teeth^35^. However, the morphological characters of the stream salamanders are very conservative, and the allozyme data do not support the validity of *B*. *taibaiensis*, which corresponds to the eastern lineage of *B*. *tibetanus* ^25^. Note that the allozyme data seem to lack sufficient variation to support the separation of *B*. *taibaiensis* and *B*. *tibetanus* ^25^. Considering the complex and controversial history of species delimitation for the genus *Batrachuperus*, we provisionally treated all Qinling Mountain samples (Fig. 1) as representatives of a single species, *B*. *tibetanus* ^22^. We employed several complementary approaches to explore the history of Qinling Mountain *B*. *tibetanus* using both molecular and morphological data. We aimed to (i) explore the phylogeographical pattern of *B*. *tibetanus* in the Qinling Mountains to test north-south and west-east distribution break hypotheses, (ii) examine the driving forces that generated the phylogeographical structure, and (iii) infer demographic history and assess the effect of glacial climate on the distribution of *B*. *tibetanus*.

## Results

### Genetic diversity, phylogenetic tree and population structure

The length of the mitochondrial *Cyt b* gene sequences used in our analysis was 814 bp located 14, 258–15, 071 of the Genbank sequence NC_008085^36^, with 111 (13.6%) nucleotide sites being polymorphic in the total data, of which 105 were parsimony-informative. No indels were observed. Among the 281 sequences in *B*. *tibetanus*, there were 37 haplotypes with their frequencies ranging from 0.4% (one sequence) to 32.7% (92 sequences). Eleven haplotypes were unique, 14 were shared among local populations. The most abundant haplotype (92 sequences) was shared by nine local populations.

**Figure 2:**
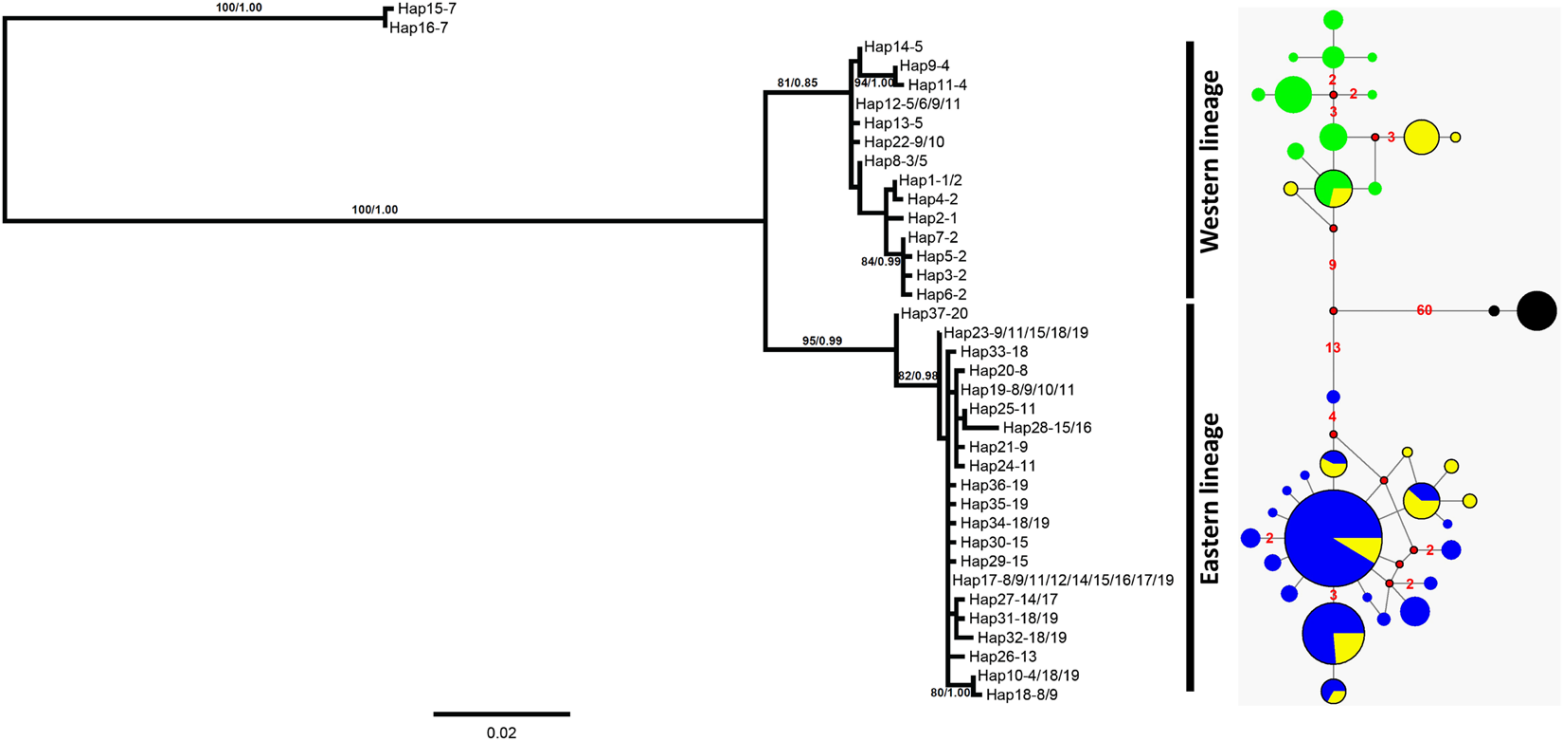
Maximum likelihood (ML) tree and the median-joining network derived from cytochrome *b* partial sequences of *B*. *tibetanus* sampled in the Qinling Mountains. The haplotypes are named by numbers (1–37). Haplotype shared by multiple locations are labelled by all location numbers separated by ‘/’. Haplotype and location numbers are separated by ‘−’. The two haplotypes (Hap15 and Hap16) from QLC population are the Tibetan lineage of *B*. *tibetanus* distributed in the Qinling Mountains (Supplementary Fig. S1), and are considered to be outgroup to explore phylogenetic relationship among the Qinling lineages. The Qinling lineages were previously described as *B*. *taibaiensis* ^35^. Numbers next to nodes of ML tree indicate bootstrap values and Bayesian posterior probabilities. Numbers in network represent the mutation steps. Sizes of circles are proportional to the haplotype frequencies. Colours for populations: green, the western group; blue, the eastern group; yellow, populations possessing haplotypes from both lineages; black, the QLC population.

All phylogenetic analyses revealed almost identical tree topologies. Thus, we only show the maximum-likelihood (ML) tree with supported values (Fig. 2). All haplotypes except for the two haplotypes from QLC population (the seventh population in Fig. 1) were clearly grouped into two major lineages separated by 23 nucleotide substitutions. With a substitution rate of 0.62% per million years^37^, the divergence time of the two lineages was about 3.5 million years ago (Ma) (95% highest posterior density, HPD: 2.2–5.0 Ma), as dated using BEAST 1.8.0^38^. We did not detect significant levels of gene flow with *N*_*e*_ *m* being 0.302 and 0.365, or less than one migrant per generation, for bidirectional migration between the two lineages using the coalescence approach as implemented in Migrate 3.5.1^39^. The two lineages were distributed in the eastern and western Qinling Mountains, respectively, and co-occurred in four populations, i.e. DP, HSP, ZH and NSG (Fig. 1). The mean pairwise difference between the two lineages was 3.70%, whereas the pairwise differences within the two lineages were 0.27% and 0.54%, respectively. Note that the populations in which the two lineages occurred were previously described as *B*. *taibaiensis* ^35^. The two haplotypes from QLC population (16 individuals) were grouped with the Tibetan lineage of *B*. *tibetanus* (Supplementary Fig. S1), which was generally distributed in the eastern edge of the Tibetan Plateau. Thus, our study demonstrated the presence of the Tibetan lineage of *B*. *tibetanus* in the northwestern Qinling Mountains. The divergence time of the QLC and the other Qinling populations was about 10.4 Ma (95% HPD: 6.5–15.0 Ma).

For all samples (281 sequences), estimates of both haplotype diversity (*h* = 0.861) and nucleotide diversity (π = 0.0242) were high, indicating high genetic diversity of *B*. *tibetanus* in the Qinling Mountains. The genetic diversity of the four populations possessing haplotypes from both the eastern and western lineages was generally larger than the other populations (Table 1). The populations of *B*. *tibetanus* in the Qinling Mountains generally displayed significant genetic differentiation (Supplementary Table S1). Simulation results from the Spatial Analysis of Molecular Variance (SAMOVA) indicated that *F*_CT_ increased greatly from *K* = 2 to *K* = 5 and then reached a plateau at *K* = 6 (Supplementary Fig. S2). Considering the single population clusters, we selected *K* = 3 as the optimal number of population groups (*F*_CT_ = 0.849; *P* < 0.001). The western group consisted of the population 1–6, the eastern group contained the population 8–20, and the third group only contained the QLC population (Fig. 1). The Mantel test revealed a significant correlated relationship (r^2^ = 0.681, *P* < 0.001) between genetic distance and geographical distance (Supplementary Fig. S3), indicating the significant effect of isolation by distance on population structure.

**Table 1:** Sample information and genetic diversity of *B*. *tibetanus* in the Qinling Mountains.

Pop.no.	Code	Locality	Sample size	*N*	*h*	π (%)	Latitude (°N)	Longitude (°E)
1	GQP	Gaoqiaopu, Fengxian	3	2	0.667 (0.314)	0.25 (0.12)	33.707	106.792
2	YHM	Yuhuangmiao, Liuba	25	6	0.723 (0.073)	0.26 (0.03)	33.849	106.874
3	SY	Sangyuan, Liuba	5	1	0	0	33.785	107.286
4	NSG	Nanshigou, Taibai	22	3	0.558 (0.057)	1.94 (0.19)	34.004	107.316
5	XL	Xiaoling, Taibai	13	4	0.782 (0.069)	0.13 (0.02)	33.891	107.431
6	TPH	Taipinghe, Taibai	5	1	0	0	33.874	107.552
7	QLC	Qianglichuan, Taibai	16	2	0.125 (0.106)	0.02 (0.01)	34.106	107.218
8	HY	Huayang, Yangxian	13	4	0.756 (0.070)	0.31 (0.05)	33.710	107.604
9	DP	Daping, Yangxian	13	7	0.885 (0.064)	1.51 (0.41)	33.690	107.612
10	HSP	Huashuping, Yangxian	3	2	0.667 (0.314)	2.29 (1.08)	33.693	107.665
11	ZH	Zhenghe, Zhouzhi	17	6	0.801 (0.076)	0.86 (0.39)	33.783	107.767
12	HEP	Huaerping, Zhouzhi	37	1	0	0	33.830	107.814
13	HZZ	Houzhenzi, Zhouzhi	4	1	0	0	33.940	107.835
14	YZD	Yezhudang, Foping	11	2	0.436 (0.133)	0.11 (0.03)	33.692	107.847
15	PNZ	Ponianzi, Foping	28	5	0.328 (0.112)	0.09 (0.04)	33.727	107.904
16	BFZ	Banfangzi, Zhouzhi	10	2	0.356 (0.159)	0.18 (0.08)	33.782	107.923
17	QLL	Qinlingliang, Zhouzhi	10	2	0.200 (0.154)	0.05 (0.04)	33.750	107.971
18	YZL	Yaoziliang, Foping	33	6	0.284 (0.102)	0.16 (0.06)	33.697	108.040
19	HGD	Heigeda, Zhouzhi	11	8	0.927 (0.066)	0.31 (0.06)	33.723	108.050
20	PHL	Pingheliang, Ningshan	2	1	0	0	33.483	108.467

Standard deviation of *h* and π estimates are shown in parentheses. *N*, the number of haplotypes; *h*, haplotype diversity; π, nucleotide diversity.

### Demographic history

**Figure 3:**
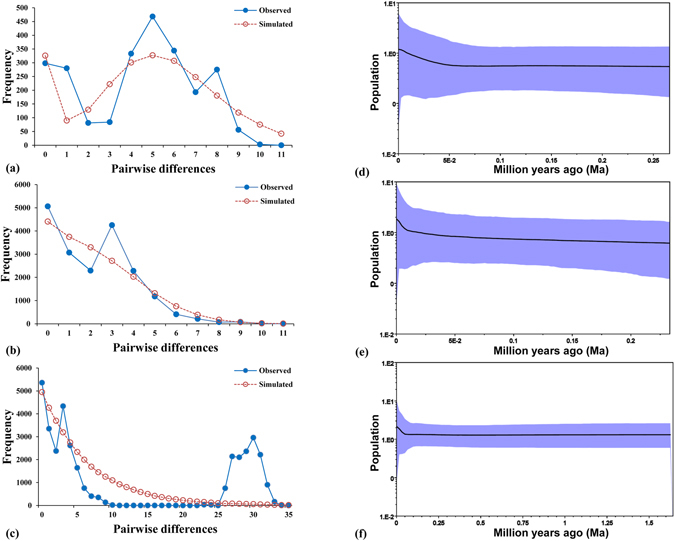
Mismatch distribution and Bayesian skyline plots (BSPs) for cytochrome *b* partial sequences of *B*. *tibetanus* sampled in the Qinling Mountains. The two approaches reveal obvious signals of demographic expansion for the Qinling lineages. The mean estimate and both 95% highest posterior density (HPD) limits are indicated. (**a**,**d**) The western lineage; (**b**,**e**) the eastern lineage; (**c**,**f**) the total samples (265 sequences).

For the eastern lineage, we detected significant evidence of demographic expansion with significantly negative values for Tajima’s *D* and Fu’s *Fs* statistics (Table 2). The mismatch distribution of the eastern lineage did not deviate significantly from the expected distribution under a sudden expansion model (Fig. 3). In contrast, the western lineage did not display any significant signals from the two summary statistics and mismatch distribution. For the two lineages, none of the statistical comparisons between the observed and simulated distributions rejected the sudden expansion model based on the raggedness index and the sum of squared deviations (SSD). In addition, positive population growth parameter (90.811 and 200.950 for the western and eastern lineages, respectively) revealed demographic expansion.

**Table 2:** Genetic diversity and demographic statistics for mitochondrial *cytb* haplotypes of *B*. *tibetanus* in the Qinling Mountains (*P < 0.05; **P < 0.01).

	n	*N*	*h*	π (%)	*g*	Tajima’s *D*	Fu’s *Fs*	SSD	Raggedness
Western lineage	70	14	0.877 (0.018)	0.54 (0.03)	90.811 (13.787)	0.495	−0.414	0.020	0.037
Eastern lineage	195	21	0.732 (0.028)	0.27 (0.02)	200.950 (16.072)	−1.494*	−7.738*	0.012	0.040
QLC	16	2	0.125 (0.106)	0.02 (0.01)	NA	−1.162	−0.700	0.042	0.578
Total	281	37	0.861 (0.016)	2.42 (0.21)	−46.661 (2.851)	0.314	6.996	0.033	0.016

Standard deviation of *h*, π and *g* estimates are shown in parentheses. n, the number of individuals; *N*, the number of haplotypes; *h*, haplotype diversity; π, nucleotide diversity; *g*, population growth parameter; SSD, sum of square deviation (goodness-of-fit to a simulated population expansion); Raggedness, raggedness index; NA, not applicable.

The coalescence-based Bayesian skyline plot (BSP) provided additional details on demographic changes through time. The western and eastern Qinling lineages of *B*. *tibetanus* displayed similar demographic history (Fig. 3). Assuming a substitution rate of 6.2 × 10^−9^ substitutions per site per year^37^, both of the two lineages showed the obvious signals of a recent expansion (50 and 10 ka for the western and eastern lineages, respectively).

### Ecological niche modelling

**Figure 4:**
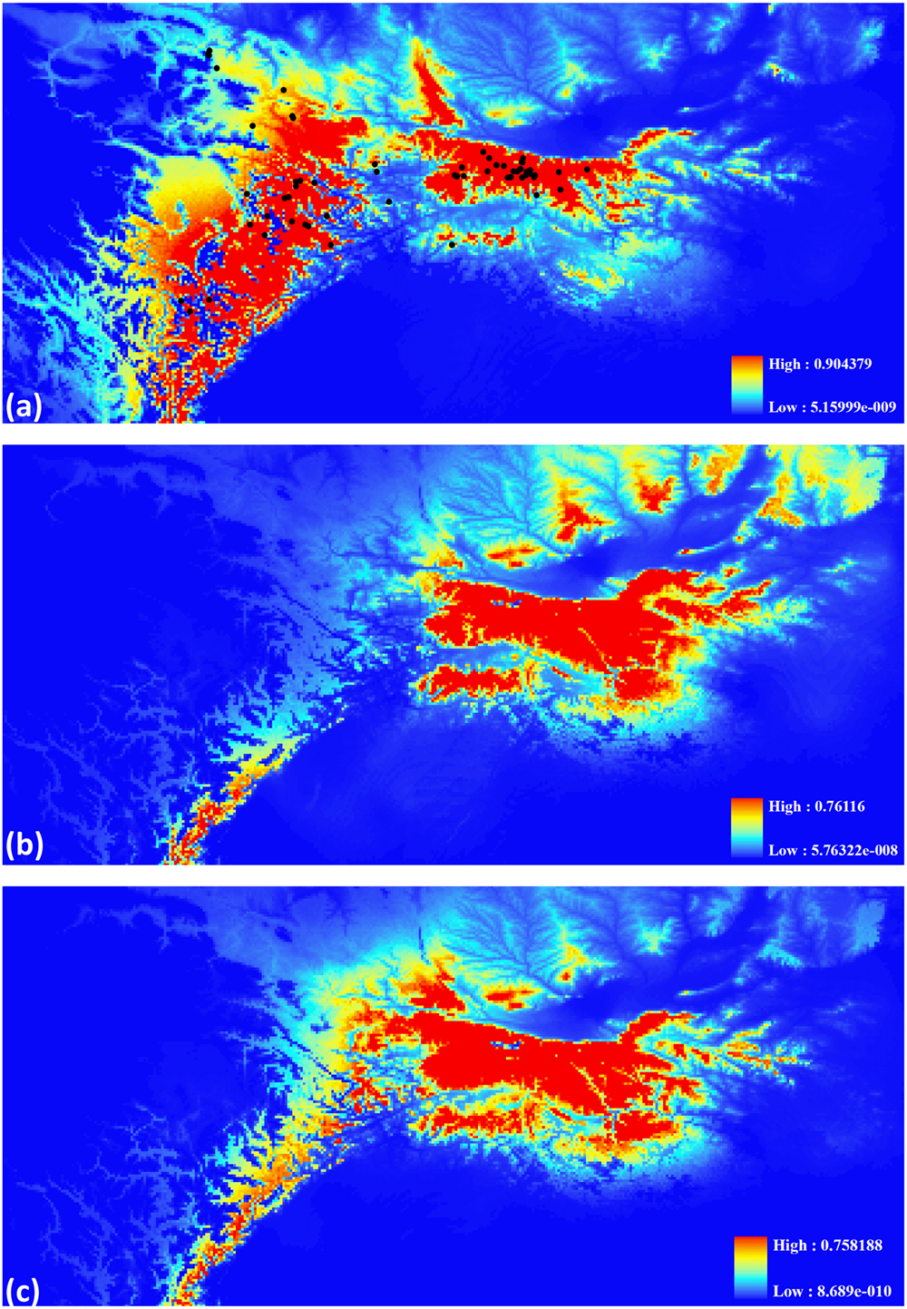
Ecological niche modelling (ENM) predicts the current (**a**) and last glacial maximum (LGM) (**b**), CSSM model; (**c**), MIROC model suitable distribution area for *B*. *tibetanus*. Circles indicate known sampling localities used in ENM analyses. The map was generated using ArcGIS 9.3 (ESRI) and Adobe Illustrator CS4 v14.0.0 (Adobe Systems Inc., San Francisco, CA).

The ecological niche modelling (ENM) predicted accurately the distribution area of *B*. *tibetanus* with the average AUC (the area under the curve) for the twenty replicate runs being 0.934, indicating a good model performance. The predicted current suitable area was markedly similar to the currently known distribution of *B*. *tibetanus* (Fig. 4a). The most important environmental variables when used alone were bio1 (Annual Mean Temperature), bio8 (Mean Temperature of Wettest Quarter) and bio10 (Mean Temperature of Warmest Quarter), according to the jackknife test. Compared to the current suitable area, the predicted past suitable area revealed a larger range in the Qinling Mountains and smaller in the eastern edge of the Tibetan Plateau at the last glacial maximum (LGM, 21 ka) (Fig. 4).

We not only included the distribution area of the Tibetan lineage of *B*. *tibetanus*, i.e. the eastern edge of the Tibetan Plateau, in our ENM analyses, but also tested the differences in habitat suitability between the Qinling and Tibetan lineages as well as the eastern and western Qinling lineages using ENMTools^40^. The identity test indicated that the predicted habitat suitability of the Tibetan and Qinling lineages of *B*. *tibetanus* exhibited statistically significant ecological differences (*P* < 0.01). The observed values for *I* (0.62) and *D* (0.32) were significantly lower than those generated from replicates. However, there was not an environmental transition between the distribution ranges of the Tibetan and Qinling lineages revealed by the range-break test (*P* < 0.68). For the western and eastern lineages in the Qinling Mountains, the differences between the habitat suitability were not significant (*P* = 0.07) with the observed values for *I* and *D* being 0.59 and 0.31, respectively.

### Analysis of morphological data

A morphological comparison (analysis of covariance) between different categories using 13 variable features is illustrated in Table 3. Three morphological variables (snout length, eye diameter and axilla-groin length) showed significant differences in mean values between the western and eastern Qinling Mountains when corrected for body size (snout-vent length as the covariate), while four morphological variables (tail length, trunk length, tail height and forelimb length) displayed significant differences between sexes. Note that the differences between the two population groups detected in this study do not greatly modify the results of the analysis of covariance. Interestingly, the factor analysis showed different trends between the four mixed populations (DP, HSP, ZH and NSG) possessing the haplotypes from the two *Cytb* lineages determined by phylogenetic methods and the other populations (Supplementary Fig. S4).

**Table 3:** A comparison of morphological features of *B*. *tibetanus* from the Qinling Mountains (analysis of covariance) (*P < 0.05; **P < 0.01).

Variable	Western group	Eastern group	Categorical variable	*F*-value
	Female	Male		
Body mass (g)	27.86 (9.57)	24.55 (9.60)	group	0.22
	25.36 (9.56)	25.67 (9.85)	sex	0.40
Tail length (mm)	98.84 (16.65)	91.43 (13.44)	group	0.54
	92.10 (14.98)	95.09 (14.53)	sex	4.81*
Total length (mm)	196.14 (27.38)	183.99 (22.01)	group	0.28
	186.43 (24.71)	188.65 (23.88)	sex	3.87
Head length (mm)	26.09 (2.92)	25.01 (2.61)	group	0.26
	25.21 (2.68)	25.44 (2.81)	sex	1.99
Head width (mm)	18.74 (2.20)	18.16 (2.32)	group	1.04
	18.33 (2.34)	18.34 (2.26)	sex	0.16
Snout length (mm)	5.34 (0.67)	5.32 (0.60)	group	4.66*
	5.32 (0.60)	5.34 (0.64)	sex	0.47
Trunk length (mm)	76.17 (9.69)	71.62 (8.19)	group	1.40
	73.48 (9.51)	72.42 (8.20)	sex	4.06*
Interocular space (mm)	6.85 (0.73)	6.69 (0.82)	group	1.88
	6.74 (0.78)	6.74 (0.81)	sex	0.21
Eye diameter (mm)	3.98 (0.46)	3.62 (0.39)	group	12.86**
	3.70 (0.45)	3.75 (0.43)	sex	0.15
Tail height (mm)	12.25 (1.87)	12.21 (2.18)	group	3.16
	11.92 (2.12)	12.53 (2.03)	sex	5.23*
Forelimb length (mm)	25.09 (2.69)	23.79 (2.29)	group	0.86
	23.89 (2.32)	24.45 (2.61)	sex	5.92*
Hind limb length (mm)	30.01 (3.62)	29.20 (2.87)	group	1.81
	29.23 (3.20)	29.65 (3.04)	sex	2.99
Axilla-groin length (mm)	52.90 (6.87)	48.12 (7.25)	group	5.27*
	50.00 (6.71)	49.02 (8.13)	sex	1.10

The dataset contained 35 individuals from the western group and 85 from the eastern group, according to the population group determined by the Spatial Analysis of Molecular Variance (SAMOVA). The measurements of morphological features in this table were mean values and standard deviation.

## Discussion

Our study revealed three lineages of *B*. *tibetanus* in the Qinling Mountains. The two major lineages were distributed in the eastern and western Qinling Mountains, respectively, and co-occurring in the middle of the Qinling Mountains (Fig. 1). We also detected the Tibetan lineage of *B*. *tibetanus* in the northwestern Qinling Mountains (Fig. 1, Supplementary Fig. S1), which has deep divergence from the two Qinling lineages (10.37% of the mean pairwise difference). The clear genetic divergence was consistently supported by the phylogenetic tree, network and SAMOVA analyses using mtDNA (Fig. 2).

Moreover, three morphological variables, i.e. snout length, eye diameter and axilla-groin length, showed significant differences between the individuals collected from the eastern and western Qinling Mountains. The morphological variables may represent adaptations to variation in environment and climate throughout their distribution regions. The possible relationship between the two morphological variables i.e. eye diameter and axilla-groin length, and the habitats of different populations was also detected in the other salamanders^41^.

Thus, the hypothesis of the drainage system’s influence on genetic patterns of *B*. *tibetanus* in the Qinling Mountains was not supported by molecular data. The drainage systems of some rivers flow from west to east (e.g. Wei River, Han River and Dan River) and therefore would contribute to north-south phylogeographical structure, which we did not observe. Instead, rivers do not appear to have acted as barriers to gene flow among the Qinling lineages of *B*. *tibetanus*, which is consistent with the previous result that divergence of *Batrachuperus* species was not correlated with river drainage system^25, 42^.

Also, the distinct climates and habitats at the south and north slopes of the Qinling Mountains seem to have little impact on the divergence of the species of *B*. *tibetanus*. Note that there were some lineages grouped with the Qinling lineages (Supplementary Fig. S1). These lineages were distributed in the other mountains of the Gansu and Sichuan provinces^25^, and thus had shallow divergence with the Qinling lineages due to geographical isolation. However, we focused on the Qinling Mountains in the Shaanxi province in this article.

Given that the main phylogeographical patterns are along the west-east axis, we speculate that historical isolation due to orogenesis of the Qinling Mountains during the late Cenozoic might have caused the genetic divergence between the two Qinling lineages of *B*. *tibetanus*. The contiguous geographical areas from the west to east that the species of *B*. *tibetanus* occupied comprised the eastern edge of the Tibetan Plateau and the Qinling and Daba Mountains. The most intense uplift of the Tibetan Plateau likely has profoundly changed the topographic features of the western Qinling Mountains^43^, giving rise to the high topographic gradient along the contiguous geographical areas. According to our field survey and the description of sampling locations in prior study^25^, the Tibet lineages of *B*. *tibetanus* were generally distributed in the high altitude localities in the west from 1800 to 3700 m above sea level, and the Qinling lineages were in the east from 1300 to 2300 m. In such circumstances, vicariance and geographical barriers are generally the significant factors yielding intraspecific and interspecific differentiation. It is reasonable that high- and low-elevation topographies and significant ecological differences between the eastern edge of the Tibetan Plateau and the Qinling Mountains (*P* < 0.01) together contributed to the deep split between the Tibet and Qinling lineages of *B*. *tibetanus*, probably occurring during the late Miocene. In contrast, orogenesis of the Qinling Mountains played a key role on the divergence of the two Qinling lineages of *B*. *tibetanus*, considering statistically non-significant ecological differences between the habitat suitability of the two Qinling lineages (*P* = 0.07) and the large suitable area since the LGM. It probably occurred during the Pliocene at 3.5 Ma, which corresponded to the orogenic period of the Qinling Mountains during the late Cenozoic. As barriers to gene flow, the Taibai Mountain (3767 m), the highest peak of the Qinling Mountains, might have contributed to the split between the two Qinling lineages.

Similar west-east and/or west-central-east breaks in the Qinling Mountains have already been commonly reported in plants^27–29^ and animals^19–21^, indicating the prevalent trend of west-east and/or west-central-east breaks in this region instead of a north-south break. Different factors appeared to play different roles on driving genetic differentiation of different species living in this region, resulting in distinct genetic differentiation patterns. For example, glacial refugia and local adaptation after dispersal probably generated a deep split in the Chinese endemic freshwater crab *Sinopotamon acutum* in the middle of the Qinling Mountains without overlap area^21^, whereas multiple lineages distributed from west to east with extremely limited overlap area detected in the frog *Feirana taihangnica*, perhaps resulted from tectonic changes and Pleistocene climatic fluctuations^20^. Our study suggests that the Qinling lineages of *B*. *tibetanus* possibly resulted from orogenesis of the Qinling Mountains and were affected at different levels by Pleistocene climatic oscillations.

The two Qinling lineages of *B*. *tibetanus* exhibited surprisingly obvious signals of demographic expansion detected by various approaches (Table 2, Fig. 3), but had different responses to Pleistocene climatic changes. The coalescence-based Bayesian skyline plot (BSP) showed that the eastern Qinling lineage experienced a long period of constant population size (0.23 Ma–0.01 Ma) followed by a postglacial expansion (about 10 kyr), which was also detected for the other amphibian species living in the Qinling Mountains, such as frogs^19, 20^. The postglacial expansion was also indicated by the most abundant haplotype (92 individuals) occurring in 9 of the 13 eastern localities and its star-like topology in the network. It suggested that the Pleistocene climatic changes might have had more impact on the demographic changes of the eastern Qinling lineage. In contrast, the western Qinling lineage experienced a long period of constant population size (0.25 Ma–0.05 Ma) followed by a recent expansion (about 50 kyr) during the last glacial epoch, indicating that the western Qinling lineage was not severely suppressed by the LGM (~21 ka). Considering restricted distribution of most haplotypes, the expansion of the two Qinling lineages probably resulted in the secondary contact in four localities with high genetic diversity, i.e. DP, HSP, ZH and NSG (Table 1).

We speculate that *B*. *tibetanus* likely experienced population expansion from the Tibet Plateau to Qinling Mountains during the late Miocene, possibly related to the recent rapid uplift of the Tibet Plateau (~10–8 Ma)^44^, considering the geographic conditions in and around the distribution areas of the Qinling lineages of *B*. *tibetanus*. First, the presence of the Tibetan lineage of *B*. *tibetanus* in the northwestern Qinling Mountains revealed in this study indicate the possible dispersal from the Tibet Plateau to the Qinling Mountains. Second, the recent rapid uplift of the Tibet Plateau potentially contributed to the expansion of *B*. *tibetanus* from the Tibet Plateau to the Qinling Mountains. The recent rapid uplift of the Tibet Plateau (~10–8 Ma) is broadly consistent with the divergence of the QLC and the other Qinling populations (95% HPD: 6.5–15.0 Ma). Dispersal from the Qinling Mountains to the Tibet Plateau was not supported in our study, such as the ENM analyses. The *B*. *tibetanus* distributed in the eastern edge of the Tibetan Plateau might be severely suppressed at the LGM (Fig. 4). In contrast, the ENM analyses revealed similar suitable areas in the Qinling Mountains since the LGM (21 ka) in the case that the two Qinling lineages show obvious signals of demographic expansion. It might be because there were not suitable montane stream habitats for expansion surrounding the Qinling and Daba Mountains, and there was significant limitation in dispersal to high elevation localities of the Tibet Plateau.

Currently active climatic changes have significant influence on the distribution of species^45, 46^. Although the amphibians (frogs, salamanders and caecilians) survived through the past great mass extinctions, many species are currently at risk globally due to various factors such as rapid ecological and climatic changes^47–50^. Habitat loss, fragmentation and over-hunting might cause reductions in population size of *B*. *tibetanus*, and then decrease genetic diversity within and among populations^51^. In this circumstance, it is important to assess genetic diversity of *B*. *tibetanus* for designing conservation strategies. *B*. *tibetanus* in the Qinling Mountains has maintained a high level of total genetic diversity with *h* and π being 0.861 and 0.0242, respectively, and exhibited surprisingly obvious signals of recent demographic expansion (Table 2, Fig. 3). Our ENM analyses indicate that *B*. *tibetanus* in the Qinling Mountains has the adaptability in the face of environmental changes (Fig. 4). These results imply a favourable status of this species for conservation and generally healthy natural environment in the Qinling Mountains. By contrast, more than 43% of amphibian species across the globe are in a state of decline^47^. However, our study revealed low genetic diversity within populations and high genetic differentiation among populations (Table 1, Supplementary Table S1), possibly resulting from habitat fragmentation, long-term and persistent human disturbance or the fluctuations in population size during the evolutionary history. It indicates the importance and urgency of preserving the populations of this species.

The past and current connectivity between populations of *B*. *tibetanus* revealed by genetic analyses provide vital information for conservation planning in the Qinling Mountains. Considering the significant genetic divergence and morphological differences between the two Qinling lineages of *B*. *tibetanus*, we suggest that the two main lineages should be considered as separate evolutionary significant units (ESU) in future conservation strategies. Our intraspecific units identified here do not meet Moritz’s strict criteria for ESU, which require both significant divergence at nuclear loci and reciprocal monophyly of mtDNA^52^. Given that the allozyme data seem to lack sufficient variation to identify the lineages corresponding to mtDNA units^25^, additional research employing polymorphic nuclear DNA markers, such as non-coding nuclear sequences^53^, may clarify it. However, the two genetic units of *B*. *tibetanus* are supported by our morphological data and meet Fraser and Bernatchez’s criterion of a lineage demonstrating highly restricted gene flow from other such lineages within the higher organizational levels (lineages) of the species^54^. The three lineages in the Qinling Mountains generally have low genetic diversity and thus deserve more surveillance for protection, especially for the QLC population and the eastern lineage (Table 2). For protecting the extremely rare animals such as the giant panda and the crested ibis (*Nipponia nippon*), a number of nature reserves (e.g. Foping National Nature Reserve) have been established in the eastern Qinling Mountains during the past fifty years. Tree cutting and hunting are prohibited in these areas, and the habitat of *B*. *tibetanus* in the nature reserves is generally in a favourable condition. Thus we should pay more attention to habitat across the large areas outside of the nature reserves by comprehensively assessing the conservation status of habitat patches and removing anthropogenic pressures. The areas in the middle of the Qinling Mountains where the two lineages co-occur also warrant protection to increase genetic variation and to maintain adaptation and evolutionary process^55^.

## Methods

The methods were performed in accordance with the guidelines of the American Society of Ichthyologists and Herpetologists^56^. All experimental procedures and animal collection were conducted under the permits (No. IOZ14001) approved by the Committee for Animal Experiments of the Institute of Zoology, Chinese Academy of Sciences, China.

### Sample collection, DNA extraction and sequence amplification

Two hundred and eighty one individuals of *B*. *tibetanus* were collected at 20 localities of Shaanxi province across its main distribution range in the Qinling Mountains between 2008 and 2010 (Fig. 1). For each location, we collected 2 to 37 samples depending on population size (Table 1). We were limited to no more than ten individuals for six localities because of natural rarity. We also downloaded previously published sequences of *B*. *tibetanus* ^25^ for the subsequent phylogenetic analysis.

After measurements of morphological variables, the tail of each salamander individual was removed and stored in 95% ethanol for DNA analysis. Genomic DNA was isolated from muscle tissues of the tail using standard phenol-chloroform procedures^57^. The fragment of mitochondrial cytochrome b (*Cytb*) gene was amplified using designed primers (pn1F: 5′-ATTCGAAAAACTCACCCATTA-3′ and pn1R: 5′-AATGTTAAGCTGCGTTGTT-3′) based on the complete mitochondrial genome of *B*. *tibetanus* (Genbank sequence NC_008085). Amplifications were performed in 30 μl reactions containing 15 ul Taq Polymerase Enzymes mixture (Takala, Da Lian), 0.3 μM of each primer, and 100 ng of the template DNA. PCR involved 5 minutes at 94 °C, then 35 cycles of 94 °C for 30 s, 50 °C for 30 s and 72 °C for 45 s, and finally an extension at 72 °C for 10 minutes. The reaction mixture was amplified using an MBS Satellite thermal cycler (Thermo Electron Corp., USA). The amplified DNA products were purified, and automated DNA sequencing was performed on an ABI3730 with an ABI PRISM BigDye terminator Cycle Sequencing Ready Reaction Kit (Perkin Elmer Biosystems). All individuals were sequenced from both strands. Sequence data were carefully inspected by eye using BioEdit 7.1.3.0^58^, and sequences immediately next to the sequencing primer were excluded.

### Phylogenetic analyses and divergence time estimation

We reconstructed phylogenetic topologies of mtDNA haplotypes using maximum-likelihood (ML) and Bayesian inference (BI) methods. The best-fitting substitution model was determined under the Bayesian information criteria (BIC) using jModelTest 2.1.1^59^. The best-fit model of nucleotide substitution for *Cytb* fragment of *B*. *tibetanus* was TPM1uf + G. We used PhyML 3.0^60^ to perform ML analyses. The pruning and regrafting (SPR) option was used to estimate better ML tree topology. The non-parametric bootstrap with 1000 replicates was used to estimate the support for each internal branch of the ML phylogeny. We used the program MrBayes 3.2.1^61^ to perform Bayesian phylogenetic inference. Markov chains were run for 10^7^ generations with two independent runs including one cold and three heated chains. We examined two convergence diagnostics, i.e. the average standard deviation of split frequencies and the potential scale reduction factor (PSRF). As runs converged, the average standard deviation of split frequencies should be lower than 0.01 and PSRF should approach 1.0. Trees were sampled every 1000 generations, and the initial 25% of trees were discarded as burn-in. The remaining trees (15002) were used to estimate the majority-rule consensus tree and the Bayesian posterior probabilities (BPP). We performed three replicate analyses. The Qinling lineages were previously described as *B*. *taibaiensis* ^35^. Thus, haplotypes from QLC population, which were grouped with the Tibetan lineage of *B*. *tibetanus* (Supplementary Fig. S1), were used as outgroup in the phylogenetic analyses. After pruned into a 725 base-pair fragment, we also performed the phylogenetic analyses for a combined dataset containing 33 sequences from Fu & Zeng^25^ to explore phylogenetic relationship among the samples from the Qinling Mountains and the Tibetan Plateau. The best-fit model for this dataset was TPM1uf + I + G.

The divergence time between the *Cytb* lineages was estimated by a mean substitution rate method in BEAST^38^. An uncorrelated lognormal clock model was used, with a mean value of 0.0062 substitutions per site per million years based on the evolutionary rates of *Cytb* estimated in salamanders^37^. We used a coalescent tree prior modelling constant population size through time that was appropriate for sequences sampled from the same population and/or species^38^. Markov chains were run for 10^9^ generations with a sampling interval of 10^5^ generations, and the first 10% of the trees were discarded as burn-in. Convergence of the parameters sampled was checked using Tracer 1.6^62^.

Levels of gene flow between the cytb lineages were estimated by the coalescence approach in Migrate. The Bayesian search strategy comprised one long chain with 10^5^ recorded steps, a sampling interval of 100 and a burn-in of 10^8^ steps. Static heating scheme was used with four chains and temperatures that are (1, 1.5, 3 and 10^6^). Runs were repeated three times to examine consistency of estimates, and convergence was also checked using effective sample size (ESS). The effective number of immigrants per generation (*N*_*e*_ *m*) of the most likely migration model was calculated by estimated mutation-scaled population sizes (*θ*) and immigration rates (*M*), *N*_*e*_ *m*_j→i_ = *θ*_i_ × *M*_j→i_.

### Population genetic diversity and structure

Genetic diversity within populations was estimated by haplotype (*h*) and nucleotide diversity (π) using DnaSP 5.10.01^63^. Pairwise sequence differences among the haplotypes were calculated using Arlequin 3.5.1.2^64^. The exact test of population differentiation was performed using Arlequin. Isolation by distance (IBD) was examined by testing the relationship between pairwise population genetic distances and geographical distances using Mantel test in Arlequin. Genetic distances between populations used in the Mantel test were calculated by MEGA 5.2^65^. To explore general pattern of variation, a median-joining network of haplotypes was constructed using NETWORK 4.6.1.0^66^.

We used SAMOVA 2.0^67^ to define the number of population groups based on geographical and genetic distance. Without a priori structure parameters, the method was based on a simulated annealing procedure to maximize the proportion of total genetic variance (*F*_CT_) due to differences between population groups. For each user-defined number of groups (*K*), *F*_CT_ was estimated and evaluated to select the optimal number of genetic groups. The number of initial conditions was set to 100 with *K* = 2–15. Statistical significance was tested using 1000 random permutations of the data.

### Historical demography

Historical demography of the *Cytb* lineages was investigated by summary statistics Tajima’s *D* ^68^ and Fu’s *Fs* ^69^ using DnaSP. Significantly negative values of Tajima’s *D* and Fu’s *Fs* are evidence for an excess of rare variations potentially due to population growth. We performed the mismatch distribution analysis to detect recent population expansion by comparing the distribution of the number of pairwise differences between haplotypes and their theoretical distribution expected under a model of sudden (stepwise) demographic expansion. The SSD and raggedness index^70^ were used to test goodness of fit. We also used a coalescent-based method in LAMARC 2.1.8^71^ to estimate the exponential growth rate (*g*) for each *Cytb* lineage and total samples. The Bayesian search strategy comprised 10 short chains of 5000 steps and two long chains of 200,000 steps, and a sampling interval of 20. Runs were repeated ten times to examine consistency of estimates. Finally, we estimated past population dynamics using coalescence-based Bayesian skyline plots^72^, as implemented in BEAST. The best-fit model with an uncorrelated lognormal clock was used. Markov chains were run for 10^8^ generations with a sampling interval of 10^4^ generations, and the first 10% of the trees were discarded as burn-in. The Tracer was used to summarize the results and check convergence of the Markov chain.

### Ecological niche modelling

We performed ecological niche modelling (ENM) to evaluate the present suitable area of *B*. *tibetanus* and to infer its potential distribution during the Quaternary (21 kya) using climate data and known sampling localities of this species. We used two models for the LGM (21 kya): the Community Climate System Model (CCSM) and the Model for Interdisciplinary Research on Climate (MIROC), originally developed from the Paleoclimate Modelling Intercomparison Project Phase II (PMIP2). Nineteen bioclimatic variables were downloaded from the WordClim website (www.worldclim.org) at 2.5 arc-min (~4 km) resolution. After downloading, we defined a study area spanned from 29.671 N to 36.536 N and from 99.420 E to 114.030 E, a larger spatial range than the current distribution range of *B*. *tibetanus*. Note that the distribution area of the Tibetan lineage of *B*. *tibetanus*, distributed in the eastern edge of the Tibetan Plateau, was included in our ENM analyses. We performed a correlation analysis between the nineteen climatic variables with ArcGIS 9.3 (ESRI) in the defined area, and then selected eleven uncorrelated variables (*r* < 0.85): bio1-Annual Mean Temperature, bio6-Min Temperature of Coldest Month, bio8-Mean Temperature of Wettest Quarter, bio9-Mean Temperature of Driest Quarter, bio10-Mean Temperature of Warmest Quarter, bio11-Mean Temperature of Coldest Quarter, bio12-Annual Precipitation, bio13-Precipitation of Wettest Month, bio14-Precipitation of Driest Month, bio17-Precipitation of Driest Quarter, bio19-Precipitation of Coldest Quarter. Species occurrence data consisted of 62 sampling localities, 32 of which were described^25^. The ENM analyses were performed using the maximum entropy algorithm in Maxent 3.3.3^73^ due to its appropriateness for presence-only data and good performance^74^. Maxent was run for twenty replicates with default parameters except for using 25% of occurrence data to test the model. We used the area under the curve (AUC) of the receiver operating characteristic (ROC) plot to evaluate model performance.

We used ENMTools 1.4.3 to perform the identity test and range-break test for testing the differences between the Qinling and Tibetan lineages as well as the Qinling lineages. The identity test is used to ask whether the predicted habitat suitability for the lineages is more different than expected if they are generated at random from a pooled data set. The range-break test used the same pooled data set to ask whether geographic boundary between the lineages is associated with an environmental transition^75^. The *I* statistic and Schoener’s *D* were used to measure overlap of predicted habitat suitability of the lineages. Both *I* and *D* ranged from 0 (completely discordant niches) to 1 (identical niches). Statistical significance was tested using 100 replicates simulated in ENMTools.

### Morphological data

The following fourteen morphological variables were examined on 120 collected specimens (60 males and 60 females) with vernier calipers to the nearest 0.1 mm: body mass, snout-vent length, tail length, total length, head length, head width, snout length, trunk length, interocular space, eye diameter, tail height, forelimb length, hind limb length, axilla-groin length. Measurements were taken from adults, as determined by the body size and weight. These morphological variables, with snout-vent length being a covariate variable to avoid the impact of body size, were analysed using analysis of covariance by SPSS 13.0. This method examined whether means of a variable are equal across different groups taking into account variability of other variables. Our analysis of covariance considered two categorical variables, i.e. population group and sex, and one covariate (snout-vent length). Here, we considered the differences between the two population groups determined by phylogenetic methods and SAMOVA, respectively.

We also used a multivariate technique, i.e. factor analysis, to describe variability among the fourteen morphological variables. It was used to visualize the distance matrix generated from morphological data. We used principal-component analysis to compute a distance matrix, and varimax rotation to minimize the complexity of the components.

### Data Availability

The sequences of cytochrome b (*Cyt b*) gene of the stream salamanders in the Qinling Mountains are available in the Genbank (The accession numbers: KX548391-KX548427).

## Electronic supplementary material

Supplementary Info
