# Supplementary material for: Phylogeographical structure and demographic expansion in the endemic alpine stream salamander (Hynobiidae: Batrachuperus) of the Qinling Mountains
Source: Sci Rep. 2017 May 12;7:1871. doi: 10.1038/s41598-017-01799-w (PMC5431969; doi:10.1038/s41598-017-01799-w)
Supplement: Supplementary file 1 — Supplementary Info [file 41598_2017_1799_MOESM1_ESM.pdf]

Scientific Reports

SUPPLEMENTARY INFORMATION

Phylogeographical structure and demographic expansion in the endemic alpine stream salamander (Hynobiidae: *Batrachuperus*) of the Qinling Mountains

Zu-Shi Huang, Feng-Lan Yu, Hui-Sheng Gong, Yan-Ling Song, Zhi-Gao Zeng and Qiong Zhang

Supplementary Table S1 Exact test of population differentiation and model-corrected average pairwise difference within and between populations of *B. tibetanus* in the Qinling Mountains (**P* < 0.05; ***P* < 0.01; ****P* < 0.001). Below diagonal, exact test; diagonal (indicated in italic), sequence divergence within population; above diagonal, sequence divergence between populations. NS, not significant.

	YHM	NSG	XL	QLC	HY	DP	ZH	HEP	YZD	PNZ	BFZ	QLL	YZL	HGD
YHM	*2.193*	10.105***	4.443***	151.638***	38.083***	24.095***	30.691***	38.093***	40.046***	38.116***	38.016***	38.219***	39.108***	38.841***
NSG	***	*20.837*	7.113***	166.958***	13.592***	5.886*	9.752***	14.132***	15.735***	14.158***	14.141***	14.211***	13.580***	14.418***
XL	***	***	*1.066*	150.048***	34.779***	20.409***	27.208***	34.835***	36.700***	34.843***	34.734***	34.949***	35.799***	35.533***
QLC	***	***	***	*0.126*	185.484***	168.111***	172.010***	180.563***	178.803***	180.521***	180.475***	180.179***	191.253***	182.108***
HY	***	***	***	***	*2.624*	1.165	0.501	0.500***	1.572***	0.522***	0.616**	0.505*	1.109***	0.440*
DP	***	***	**	***	NS	*15.734*	-0.241	1.556***	2.809***	1.569***	1.621	1.586	2.803***	1.715
ZH	***	***	***	***	*	NS	*8.928*	0.328***	1.474***	0.321**	0.3814	0.344	2.397***	0.463
HEP	***	***	***	***	***	***	***	*0*	1.033***	0.010	0.092*	0	2.191***	0.077***
YZD	***	***	***	***	***	***	***	***	*0.885*	0.995***	0.984***	0.738**	3.193***	0.860**
PNZ	***	***	***	***	***	***	***	*	***	*0.787*	-0.014	0.004	2.213***	0.076*
BFZ	***	***	***	***	***	***	NS	*	**	NS	*1.471*	0.072	2.308***	0.148
QLL	***	***	***	***	**	***	NS	NS	**	NS	NS	*0.406*	2.187***	0.043
YZL	***	***	***	***	***	***	***	***	***	***	***	***	*1.326*	1.787***
HGD	***	***	***	***	***	**	**	***	***	***	***	***	***	*2.624*

Supplementary Table S2 X/Y coordinates of presences of *B. tibetanus* in China used for ecological niche modelling (ENM).

x	y
35.706	102.760
35.683	102.779
35.754	102.787
34.106	107.218
33.780	105.493
33.904	105.464
34.659	104.139
34.690	104.129
35.117	103.983
35.470	102.906
34.533	103.486
33.617	104.183
32.967	104.122
33.349	104.001
33.370	104.061
32.917	104.333
32.892	104.387
32.583	104.750
33.633	104.250
33.602	104.485
33.550	104.183
32.920	103.442
33.061	103.722
32.743	103.681
33.416	103.390
31.690	102.780
31.501	102.469
31.666	102.321

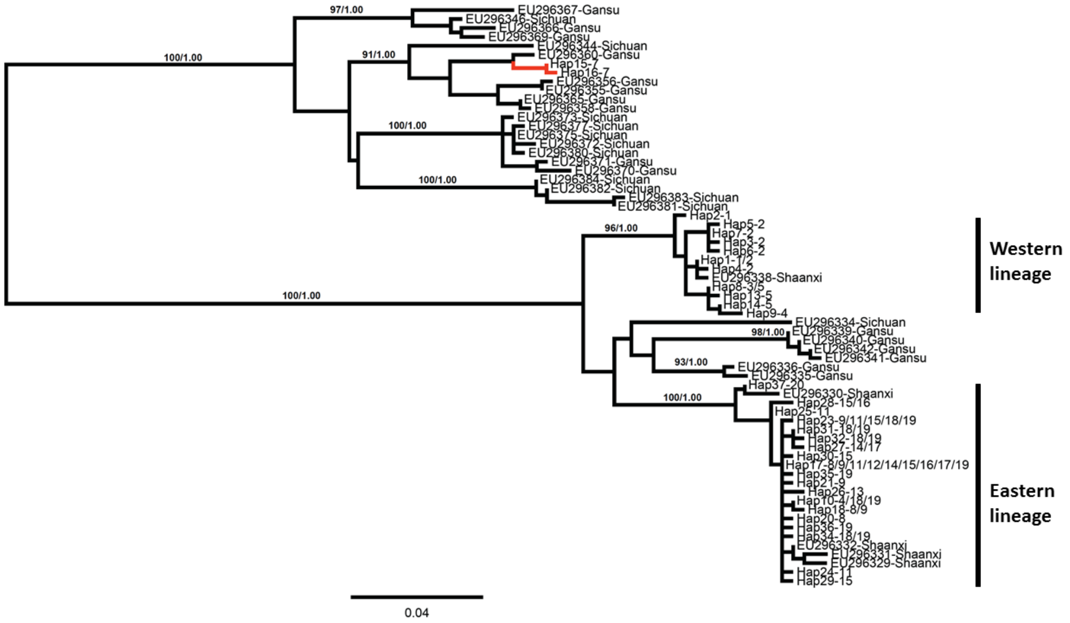

Supplementary Fig. S1 Maximum likelihood tree derived from cytochrome *b* partial sequences of *B. tibetanus*. The sequences labelled with Genbank accession numbers and geographic sources are previously published in Fu & Zeng (2008) [25]. The haplotypes used in our study are named by numbers (1-37). Haplotype shared by multiple locations are labelled by all location numbers separated by '/'. Haplotype and location numbers are separated by '-'. The two haplotypes (Hap15 and Hap16) from QLC population are the Tibetan lineage of *B. tibetanus* distributed in the Qinling Mountains. The haplotypes grouped with the eastern and western Qinling lineages are distributed in the Gansu and Sichuan provinces. These lineages except for the Tibetan lineage are previously described as *B. taibaiensis*[35]. Numbers next to nodes indicate bootstrap values and Bayesian posterior probabilities.

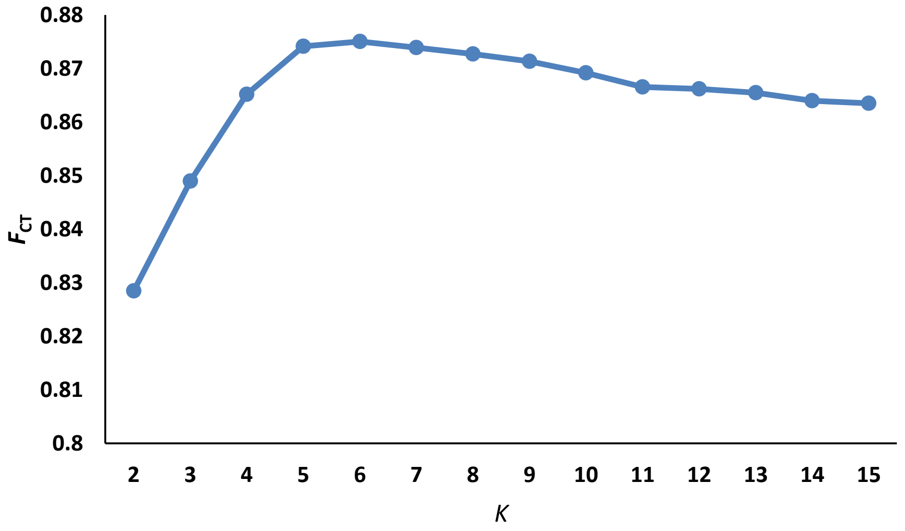

Supplementary Fig. S2 Fixation index (F_{CT}) as a function of the user-defined number of groups of populations *(K) f*or 20 of *B. tibetanus* in the Qinling Mountains using SAMOVA.

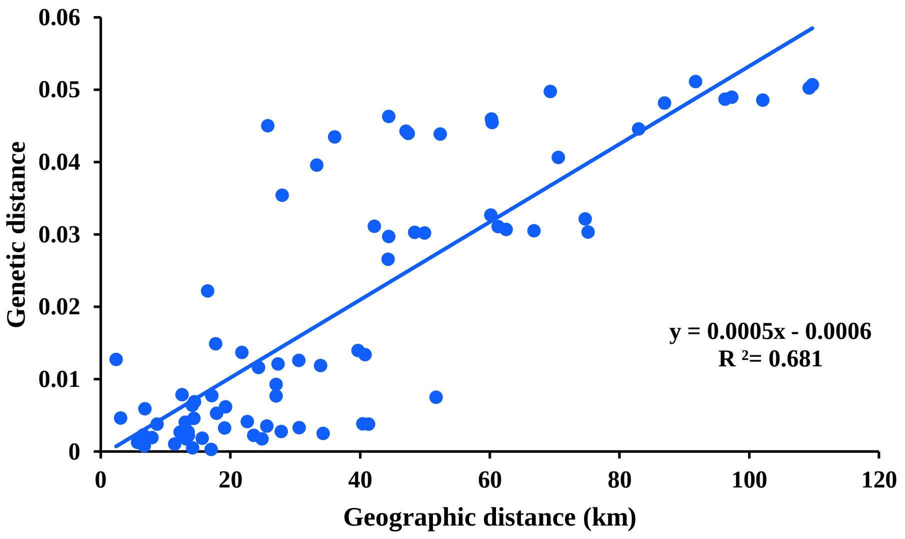

Supplementary Fig. S3 Results of Mantel tests show significant correlation between genetic distance and geographical distance (r^2 =0.681, $P < 0.001$), corresponding with the hypothesis of isolation-by-distance model (IBD) (13 populations with no less than 10 samples were involved).

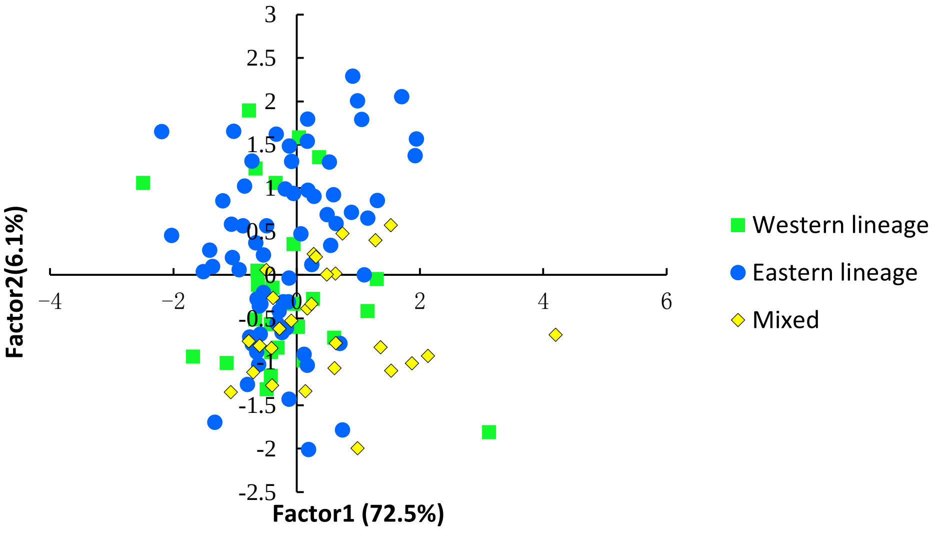

Supplementary Fig. S4 Factor analysis based on morphological data. The individuals from the western and eastern lineages are labelled by square and circle, respectively. The individuals from four mixed populations (i.e. DP, HSP, ZH and NSG) possessing the haplotypes from the two *Cytb* lineages are labelled by diamonds.
